# Imatinib dose escalation versus sunitinib as a second line treatment in KIT exon 11 mutated GIST: a retrospective analysis

**DOI:** 10.18632/oncotarget.5136

**Published:** 2015-09-26

**Authors:** Bruno Vincenzi, Margherita Nannini, Elena Fumagalli, Giuseppe Bronte, Anna Maria Frezza, Delia De Lisi, Mariella Spalato Ceruso, Daniele Santini, Giuseppe Badalamenti, Maria Abbondanza Pantaleo, Antonio Russo, Angelo Paolo Dei Tos, Paolo Casali, Giuseppe Tonini

**Affiliations:** ^1^ Department of Oncology, University Campus Bio-Medico, Rome, Italy; ^2^ Department of Specialized, Experimental and Diagnostic Medicine, Sant'Orsola-Malpighi Hospital, University of Bologna, Bologna, Italy; ^3^ Adult Mesenchymal Tumor Medical Oncology Unit, Fondazione IRCCS Istituto Nazionale Tumori, Milan, Italy; ^4^ Section of Medical Oncology, Department of Surgical, Oncological and Oral Sciences, University of Palermo, Palermo, Italy; ^5^ Department of Oncology and Pathology, General Hospital of Treviso, Treviso, Italy

**Keywords:** imatinib, sunitinib, second line, GIST, exon 11

## Abstract

We retrospectively reviewed data from 123 patients (KIT exon 11 mutated) who received sunitinib or dose-escalated imatinib as second line.

All patients progressed on imatinib (400 mg/die) and received a second line treatment with imatinib (800 mg/die) or sunitinib (50 mg/die 4 weeks on/2 off or 37.5 mg/day). Deletion versus other KIT 11 mutation was recorded, correlated with clinical benefits.

64% received imatinib, 36% sunitinib. KIT exon 11 mutation was available in 94 patients. With a median follow-up of 61 months, median time to progression (TTP) in patients receiving sunitinib and imatinib was 10 (95% CI 9.7–10.9) and 5 months (95% CI 3.6–6.7) respectively (*P* = 0.012). No difference was found in overall survival (OS) (*P* = 0.883). In imatinib arm, KIT exon 11 deletions was associated with a shorter TTP (7 vs 17 months; *P* = 0.02), with a trend in OS (54 vs 71 months *P* = 0.063). No difference was found in patients treated with sunitinib (*P* = 0.370).

A second line with sunitinib was associated with an improved TTP in KIT exon 11 mutated patients progressing on imatinib 400 mg/die. Deletions in exon 11 seemed to be correlated with worse outcome in patients receiving imatinib-based second line.

## INTRODUCTION

GIST account for 0.1–3% of gastrointestinal (GI) malignant tumours and represent the most common mesenchymal tumour of the GI tract (80%). Approximately 50% of GIST occur in the stomach, 30% are detected in the jejunum or ileum, 5% in the duodenum, 5% in the rectum and less than 1% in the oesophagus [1].

About 85–95% of GIST harbour oncogenic mutations affecting KIT or PDGFRA, two highly homologues cell surface tyrosine kinase receptors for stem cells and platelet-derived growth factor. KIT is involved in more than 75–85% of cases: almost 85% of KIT mutations occur in the juxtamembrane domain (exon 11) and the 15% in the extracellular domain (exon 9) [2]. The majority of PDGFRA mutations affect the TK2 domain (exon 18) [3]. Since their identification as a separate entity in the late 1990s, GIST were observed to be highly insensitive to standard chemotherapy and metastatic GIST were regarded as an incurable disease. As soon as GIST pathogenesis and progression was found to be driven by a KIT or PDGFRA mutations, they became the ideal solid tumour model for tyrosine kinase inhibition therapies. The introduction of imatinib mesylate (Glivec^®^), a small-molecule tyrosine kinase inhibitor active against BCR-ABL, KIT, and PDGFR, changed dramatically the prognosis of unresectable or metastatic/advanced GIST patients, achieving a disease response in more than 50% of treated cases and stabilisation in almost 30%. On the basis of these encouraging results, imatinib was licensed in February 2002 as a first line treatment for unresectable and/or metastatic GIST at the dose of 400 mg daily [4]. Subsequently, a large retrospective analysis from the MetaGIST group proved an advantage in progression-free survival (PFS) for KIT exon 9 mutated patients receiving imatinib 800 mg daily upfront, suggesting that a higher dose should be consider in this subgroup. Moreover, a dose escalation to 800 mg/day at the time of progression on first-line imatinib 400 mg/day was proven to be associated with new disease stabilisation in almost 30% of cases [5]. Sunitinib malate (Sutent^®^) is an oral multitargeted receptor tyrosine kinase inhibitor with selectivity for KIT and PDGFRA (and for PDGFRB, all three isoforms of vascular endothelial growth factor receptor, FMS-like tyrosine kinase 3, colony-stimulating factor 1 receptor and glial cell line-derived neurotrophic factor receptor) [6], whose administration is associated with a significant improvement in PFS (27.6 vs 6.4 weeks with placebo, *P* < 0.0001) for metastatic GIST patients progressing on imatinib. In 2006, FDA granted approval for sunitinib malate for the treatment of GIST after disease progression on (or intolerance to) imatinib, at the dose of 50 mg, 4 weeks on and 2 weeks off treatment [7]. The continuous daily dosing of sunitinib at 37.5 daily is an active alternative dosing strategy with favourable safety [8].

In GIST patients progressing on sunitinib, regorafenib can provide a significant improvement in PFS compared with placebo. Based on the results from a randomized, phase III study, in July 2013 FDA approved oral regoarefenib (Stivarga ^®^) as a third line treatment [9].

Up today the best choice in second line is still unclear, being both imatinib dose escalation and sunitinib reasonable options. This is particularly true for KIT exon 11 mutated patients, given their excellent response to 400 mg in first line.

Aim of the present retrospective study is to compare the outcome of metastatic GIST patients harbouring KIT exon 11 mutations treated with sunitinib versus dose-escalated imatinib after progression on imatinib 400 mg/die.

## RESULTS

### Patients population features

Patients characteristics are summarised in Table 1. Median age in our population was 58 years (range: 35–81). Sixty-eight (55%) patients were male and 55 (45%) were female.

**Table 1:** Patients characteristics

	Number of patients	% of patients
Gender (Male)	68	55%
Gender (Female)	55	45%
Age, years		
Median	58	
Range	35–81	
PS^1^ (ECOG 0 −1)	102	82%
Primary tumor Stomach	71	57%
Small bowel	31	25%
Colon rectum	21	18%
Primary tumors not resected at diagnosis	48	39%
Liver involvement	71	58%
>2 disease sites	39	32%
Peritoneal involvement	43	35%
Adjuvant imatinib	26	21%
Second line with Sunitinib	44	36%
Second line with Sunitinib alterantive schedule	16	13%
Second line with Imatinib (800 mg)	79	64%
Gist with exon 11 mutation detected	94	76%
Deleted exon 11	42	34%
Other exon 11 mutation	52	66%
Imatinibintolerance	0	

1) Performance status

One-hundred-two out of 123 subjects had an Eastern Cooperative Oncology Group (ECOG) performance score of 0 or 1.

The site of primary GIST was stomach in 71 patients (58%), small bowel in 31 (25%), colon or rectum in 21 (17%), specifically 14 localized in the colon and 7 in the rectum. The primary tumour was unresected at the beginning of treatment course in 48 patients (39%) and 71 (58%) showed liver involvement, Thirty nine patients (32%) had more than two metastatic organs involved (liver and local infiltration of other organs or peritoneal), 43 (35%) patients showed peritoneal involvement.

The specific subtype of KIT exon 11 mutation was available for 94 (76%) patients: 42 (34%) harboured a KIT exon 11 deletion, while in 52 patients (66%) different exon 11 genomic aberrations were detected.

Twenty-six (21%) patients received adjuvant imatinib after surgery, in 17 patients for the duration of one year, in the remaining 9 patients for three years. When progression occurs at the end of adjuvant treatment, all patient received imatinib 400 mg daily as firs line treatment.

The median duration of imatinib fist line therapy was 31 months (95% CI: 6–69 months). After progression on imatinib, 79 patients (64%) received imatinib dose escalation, 44 (36%) sunitinib (28 patients were treated according to the schedule 50 mg 4 weeks on/2 weeks off; 16 patients were treated with 37.5 mg/day continuous daily dosing). Patients who progressed with Imatinib dose escalation received sunitinib. Median follow-up was 61 months (95% CI: 6–124 months). No intolerant imatinib patients were included in this retrospective analysis. In our series patients reported the common toxicity described in literature both in two arms, no G3 e G4 CTCAE were reported.

### Radiological response and survival

Clinical benefit from imatinib dose escalation was observed in 46 patients (58.2%) (95% exact CI, 39.0–64.0), and among those, 15 patients (18.9%) achieved a partial radiological response.

In patients treated with sunitinib clinical benefit was observed in 29 of 44 patients (65.9; 95% exact CI, 39–79.0), and in 14 (31.8%) an objective partial radiological response was achieved.

Considering clinical benefit rate and radiological response no statistical significant difference was detected in both treatment arms (respectively, *P* = 0.5194 and *P* = 0.1660) Table 2.

**Table 2:** Clinical benefit rate according to treatment

	Patients	Clinical benefit rate	Partial radiological response
Imatinib (dose escalation)	79	58.2%	18.9%
Sunitinib	44	65.9%	31.8%
*P* values		0.5194	0.1660

**Figure 1:**
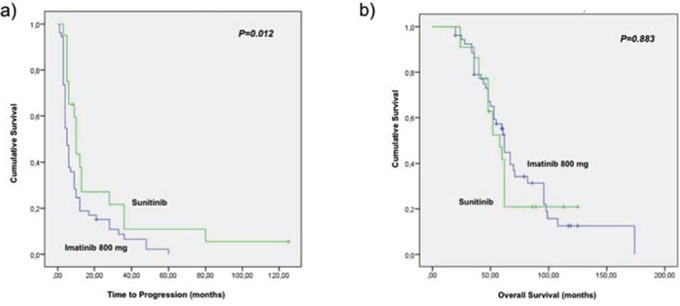
Survival results **a.** time to progression in the population treated with sunitinib as second line treatment was 10 months (95% CI 9.7–10.9) compared with 5 months (95% CI 3.6–6.7) in those who received imatinib 800 mg (*P* = 0.012). **b.** overall survival in the sunitinib and imatinib arm (58 versus 62 months respectively, *P* = 0.883).

The median time to progression (TTP) in the population treated with sunitinib as a second line treatment was 10 months (95% CI 9.7–10.9) compared with 5 months (95% CI 3.6–6.7) in those who received imatinib 800 mg (*P* = 0.012) [Figure 1a]. No significant difference was found in term of overall survival between patients treated with sunitinib and those treated with imatinib (OS) (62 versus 58 months respectively, *P* = 0.883) between the two groups of patients, since patients who progress after imatinb escalated dose were cross over to receive treatment with sunitinib. In addition, no difference was detectable between patients treated with continuous dose schedule of sunitinib in comparison with the alternate schedule 4 weeks on/2 weeks off. More in details patients treated with the continuous dose showed a median TTP of 10.3 months (95% CI 9.6–11.0) vs 9.2 months (95% CI 9.0–10.2) of patients treated with the alternate schedule (*P* = 0.801); the same in terms of OS: patients treated with the continuous dose showed a median OS of 64.6 months (95% CI 48.8–81.5) vs 60.4 months (95% CI 43.5–79.4) of patients treated with the alternate schedule (*P* = 0.764).

### Clinical outcome according to the type of KIT exon 11 mutation and in relation to the second line

Overall, considering the entire population independently by the second line choice the specific type of exon 11 KIT mutation was demonstrated to represent a significant prognostic factor in our patients' population.

In details, 11 KIT deletion compared with other exon 11 KIT mutations was identified as a negative prognostic factor in our patients population only for OS (76.0 95% CI: 68.5–114.5- vs 53.0 −95%CI: 47.9–88.4- *P* = 0.016), while there was no significant impact on Progression free survival (PFS) (9.4 −95%CI: 7.5–11.1- vs 7.8 −95% CI: 6.9–10.3- *P* = 0.261). These results confirm data from Kontogianni et al. that consider kit exon 11 deletion especially in cod 557/558 as independent prognostic factor in primary localized GIST [10].

Presence of deletions in KIT exon 11 was found to be associated with a shorter TTP in patients receiving imatinib-based second line therapy (7- 95% CI 3.1–10.4- and 17 months -95%CI 9.8–23.5- (*P* = 0.02), respectively). A negative trend was also recorded in terms of OS (54 -95%CI 21.0- 73.3- vs 71 -95%CI 45.3- 82. 8- months, (*P* = 0.063) respectively). Conversely, deletions in KIT exon 11 were not associated with a decrease activity of sunitinib in second line (TTP: 9 -95% CI 7.9–10.5- vs 12 -95%CI 9.4–13.2- months, *P* = 0.6; OS: 51 -95%CI 44.3–60.7- vs 54 -95%CI 47.8–64.3- months, *P* = 0.370). These results are summarized in Tables 3 and 4.

**Table 3:** Time to progression (months) according to type of exon 11 mutations and second-line treatment

Type of Exon 11 mutation	Imatinib Time to progression (months)	SunItinib Time to progression (months)
Exon-11 deletions	7 months	9 months
Exon-11 other mutations	17 months	12 months
*P*-Values (Time to progression)	0.02	0.683

**Table 4:** Overall survival (months) according to type of exon 11 mutations and second-line treatment

Type of Exon 11 mutation	Imatinib Overall survival (months)	Sunitinb Overall Survival (months)
Exon 11 deletions	54 months	51 months
Exon 11 other mutations	71 months	58 months
*P* values (Overall survival)	0.063	0.370

## DISCUSSION

GIST are the most common mesenchymal neoplasm of the gastrointestinal tract (80%) and represents about 5% of all sarcomas. Molecular characteristics were misdiagnosed until 1988 when Hirota and colleagues demonstrated that almost all GIST expressed KIT by immunohistochemistry (IHC) and harboured activating KIT mutations [11]. Nowadays, GIST represents a paradigm for successful targeted treatment with tyrosine kinase inhibitors (TKIs) and imatinib is regarded as the 1st-line treatment of choice in the metastatic setting. It is widely demostrated that KIT and PDGFRA mutational status affect response to imatinib. Phase II and III imatinib trials in patients with advanced GIST reported higher partial response rates and longer overall survival in patients with mutations in KIT exon 11 compared to patients with a mutation in KIT exon 9 or no detectable mutations (GIST wild-type) [12] Furthermore, almost all patients who experience early disease progression on imatinib treatment (400 mg/day) have mutations in KIT exon 9 or in PDGFRA, or are wild type for the known KIT and PDGFRA mutations [5]. However, patients with KIT exon 9 mutations have been shown to better respond to the double dose of imatinib (800 mg/day), although a higher incidence of adverse events has been reported [13]. On the basis of phase III studies showing that patients with KIT exon 9 mutations may achieve longer PFS on higher-dose imatinib [14], guidelines support the use of imatinib 800 mg/day as the standard treatment of choice in this molecular subgroup. As far as these data among patients with GIST who experienced progression on 1st-line imatinib 400 mg/day, clinical data suggest that 2nd-line treatment with either imatinib 800 mg/day or sunitinib may be considered as subsequent treatment, and those who progress on 1st-line imatinib 800 mg/day may be switched to sunitinib. The efficacy of imatinib 800 mg/day after disease progression has been investigated in two phase III clinical trials where patients with disease progression on the once daily (400 mg/day) imatinib regimen were offered the option of crossover to the twice daily (800 mg/day) arm. Both these trials showed a significant benefit in terms of progression free survival (PFS) and overll response rate (ORR) in patients who cross-over from imatinib 400 mg/day to imatinib 800 mg/day concluding that a cross-over to high-dose imatinib is feasible and safe in GIST patients who progress on low-dose therapy [13, 14]. On the other hand sunitinib administered at 50 mg/day in 6-week cycles comprising 4 weeks on treatment followed by 2 weeks off treatment (schedule 4/2) becomes a valid alternative after prior progression to imatinib 400 mg/die. In a phase III double-blind trial, patients with an imatinib-resistant/-intollerant GIST were randomized 2: 1 to receive sunitinib 50 mg/day on schedule 4/2 or placebo with cross-over from the placebo group to the sunitinib arm. The trials showed a benefit in terms of PFS and TTP in the sunitinib arm versus the placebo group [7].

Nowadays, in case of disease progression under imatinib 400 mg/die as adjuvant setting or as first-line therapy, the choice of a suitable second line therapy is still a matter of debate in clinical practice, since no data that compare imatinib 800 mg/die vs sunitinib as second line treatment are available. Moreover, considering only the KIT exon 11 mutated patients, who are the most sensitive to imatinib 400 mg, when a progression occurs, no data are available to support the use of imatinib 800 mg/die as second line treatment in comparison with sunitinib [15]. A systematic review analysing the effectiveness of Imatinib escalating dose versus Sunitinb in patients who progressed after firs t line 400 mg/daliy Imatinb was conducted by Hislpo. The authors concluded that the statistical likelihood of response on both treatment may depend on exon mutational status encouraging further clinical randomized trials [16].

To our knowledge, this is the first retrospective analysis comparing the outcome of metastatic/advanced KIT exon 11 mutated GIST patients treated with sunitinib or dose-escalated imatinib as second line treatment. The aim of this study has been to identify which subgroup of metastatic KIT exon 11 mutated GIST patient could benefit of sunitinib rather than imatinb in order to better tailor the best therapeutic choice after the first line progression on the basis of molecular status. Moreover patients were stratified according to the type of KIT exon 11 mutation (deletions versus others) in order to verify if could be a different response to the two TKIs.

Our data showed that patients harbouring exon 11 deletions present a worse outcome in terms of OS in both treatment arms comparing patients with other exon 11 mutation; highlighting a possible negative prognostic role of deletion in this patients subset.

Furhermore the results of the study showed that there is an advantage in term of TTP in patients treated with sunitinib in comparison with patients in the imatinib arm, even if no difference in term of overall survival was found, since that OS is affected by all treatment history of each patients and by the biological background in part still unknown.

Interestingly, we showed that imatinib was less effective in the subgroup of patients harbouring a KIT exon 11 deletion than in those harbouring other kind of mutation in exon 11 in term of TTP, while no difference was found in patients treated with sunitinib. The present result is apparently in contrast with the data of the phase III trial that compared sunitinib to placebo as second line treatment in patients progressing after imatinib. Although a clinical benefit of sunitinib treatment was observed in all major mutant types, the primary response rate was significantly higher for KIT exon 9 mutants than KIT 11 ones, suggesting a possible role of primary and secondary mutations on sunitinib activity [7]. In fact, the impact of primary and secondary kinase genotype on sunitinib activity was evaluated in different studied confirming that patient with primary mutations on KIT exon 9 had a longer PFS and OS than patient with KIT exon 11 mutations, while a better response was found in KIT exon 11 mutated patients with secondary mutations on exons 13 and 14 than in patients with secondary mutations on exons 17 and 18 [17, 18] [19]. These data were also confirmed *in vitro* study where GIST cell with primary exon 11 mutation who secondary acquired a KIT exon 13 mutation were more sensitive to sunitinib than imatinib [18]. According to these data, we can hypothesize that the KIT exon 11 mutated patients in our series, who better responded in term of TTP in the sunitinib arm may harbor a second acquired mutation in the exons that are particularly sensitive to sunitinib such as 13 on 14 exons, even of molecular data on secondary mutations are not available, since both Italian and European guidelines do not advise the use secondary biopsy at imatinib progression. Futhermore considering data from literature we can hypotyze that exon deletion on kit-11 in our series, presents a worse prognosis respect to not deleted GIST, confirming data from previous study that reported exon 11 mutation especially in resected gastric GIST as independent factors of worse prognosis [20, 10]. Furthermore we can suppose that these subset of patients may acquire secondary mutation resistant to imatinib escalated dose than to sunitiib as reported in our results in terms of TTP.

However, as is well known, several molecular mechanisms underlying secondary resistance in GIST have been described and in many cases the secondary resistance may not sustained by the acquisition of novel mutations [21]. Therefore, the different efficacy of sunitinib and of imatinib dose escalation in KIT mutated GIST patients as second line treatment, could be sustained by other molecular mechanisms still unknown.

In conclusion, to our knowledge, this is the first retrospective report on the comparison between imatinib 800 mg and sunitinib as second line treatment in KIT exon 11 mutated GIST patients. On the basis of our preliminary results, sunitinib as second line treatment may be mostly considered in patients with KIT exon 11 deletion after failure to imatinib. However, the clinical value of this analysis should be validated in prospective studies on larger series of patients, in order to tailor the choice of second line treatment according to the molecular data. Moreover, the repetition of the mutational analysis with new diagnostic tool such liquid biopsy [22], if feasible, after disease progression, may be recommended in order to identify secondary mutations or other alterations and choose the best second line option in accordance to their sensitivity to TKIs.

## MATERIALS AND METHODS

### Patients

One-hundred-twenty-three consecutive metastatic or advanced GIST patients treated at four referring Italian institutions (University Campus Bio-Medico, Rome; Sant'Orsola Malpighi Hospital, Bologna; Istituto Nazionale Tumori, Milan; Policlinico P. Giaccone, Palemo) were included in the present retrospective analysis. All the patients included harboured a KIT exon 11 mutation and progressed after a first line with imatinib 400 mg/die. Up on physician choice, they all receive either imatinib dose escalation (800 mg/die) or sunitinib (50 mg/die 4 weeks on/2 weeks off or 37.5 mg/die continuous daily dosing) as second line treatment. There were no difference in terms of age, gender and second line treatment causes between two arms even if it was a retrospective analysis. KIT mutational analysisis was made at the diagnosis. In patient with Gist resected at primary diagnosis the mutational status was detected on the primary site. All the analysis was made with polymerase chain reaction (PCR) amplification of genomic DNA.

Methods used for the analysis were carried out in accordance with the approved guidelines, and the institutional approval committee of Campus Bio-Medico of Rome approved the experimental protocol.

### Data collection

A database including details on patient's demographic characteristics (age, gender), disease (site of the primary, presence of the primary, peritoneal involvement liver involvement and type of KIT exon 11 mutation), treatment (imatinib dose escalation or sunitinib), response to the treatment and outcome was shared among the contributing centres and data were retrospectively collected. Response to treatment defined as Clinical Benefit Rate (percentage of patients who have achieved complete response, partial response and stable disease) was evaluated according to RECIST 1.1 criteria or according to Choi criteria (on the basis of physicians' experience), since this was a retrospective analysis no central radiological review was made.

### Statistical analysis

Descriptive analysis was made using median values and range. Differences between groups were assessed using the Chi-squared test. Time to progression (TTP) was calculated as the period from the treatment start to the first evidence of disease progression. Overall survival (OS) was calculated from the time of diagnosis of metastatic GIST to the time of death or the last documented time the patient was known to be alive. Patients with no evidence of progression were censored at the last tumor assessment. Death was considered an event regardless of the cause. Patients alive or lost to follow-up were censored at the last contact. Survival analysis was performed by Kaplan-Meier product-limit method and the differences in term of TTP and OS according to the treatment received or the type of mutation detected were evaluated by the log-rank test. SPSS software (version 17.00, SPSS, Chicago, ILQ5) was used for statistical analysis. A *P* value of less than 0.05 was considered to indicate statistical significance.
